# Risk factors for *Ascaris lumbricoides* infection and its association with nutritional status and IQ in 14-Year old adolescents in Chitwan, Nepal

**DOI:** 10.1038/s41598-024-77306-9

**Published:** 2024-10-29

**Authors:** Rajendra Prasad Parajuli, Shristi Bhandari, Lauren Marie Ward, Jose Ricardo Suarez-Lopez

**Affiliations:** 1 Central Department of Zoology, Tribhuvan University, Kritipur, Kathmandu Nepal; 2 Herbert Wertheim School of Public Health and Human Longevity Science, University of California San Diego (UCSD), CA USA San Diego,; 3 Dept. of Environmental Health Sciences, University of Michigan School of Public Health, Washington Heights, Ann Arbor, MI 1415, 48109 USA

**Keywords:** *Ascaris lumbricoides*, Intelligent Quotient (IQ), Nutritional status, ACE score, Nepal, Social determinants of health, Developmental biology, Neuroscience, Zoology, Environmental sciences, Environmental social sciences, Diseases, Risk factors

## Abstract

Inconsistent results have been obtained from studies investigating the association between soil-transmitted helminths (STHs) and nutritional status and cognitive outcomes. This study aimed to investigate the influence of STHs and nutritional status on the intelligence quotient (IQ) of adolescents in the Chitwan Valley, Nepal. In this birth cohort study from the Chitwan district of Nepal, 74 cohort participants were followed up after 14 years. The presence and egg density of the STHs were investigated following the Kato–Katz method. Full-scale IQ was evaluated using the Wechsler Abbreviated Scale of Intelligence-II (WASI-II). Multivariate regression analysis was also conducted to examine the associations of STHs and nutritional status with IQ scores, adjusting for adverse childhood experiences (ACE) and demographic and socioeconomic factors. The only STHs detected was for *Ascaris lumbricoides*, which was detected in 16% (12/74) of the study participants. Similarly, 16% (12/73) of participants reported undernutrition (body mass index (BMI) Z score<-2SD), and 33% (25/74) of participants had a developmental deficit (WASI II FSIQ < 69). There were no significant associations between the presence of *A. lumbricoides* or egg density and IQ scores. However, undernutrition (BMIZ<-2SD) increased the odds of a developmental deficit according to both univariate and adjusted multivariate models [i.e., adjusted odds ratio (aOR) 9.41; 95% CI (2.23 to 39.80)]. While the study primarily examines the association of STHs and nutritional status with IQ, it is important to note that adolescents with educated mothers or higher incomes also showed better cognitive development. The prevalence of undernutrition and associated developmental deficits characterized by an IQ < 69 was alarmingly high in this cohort of adolescents from Chitwan, Nepal. Despite the high prevalence of *A. lumbricoides*, its infection was not identified as significantly associated with cognition. However, it is noteworthy that a consistent favorable impact of an enriching postnatal environment, approximated by maternal education and family income, may improve neurodevelopmental indicators.

**Supplementary Information:**

The online version contains supplementary material available at 10.1038/s41598-024-77306-9.

## Introduction

Intestinal parasitosis affects approximately 1.5 billion people worldwide, including 654 million school-age children^1^. Overall, the most common intestinal parasitic infections (IPIs) are *Ascaris lumbricoides*, *Trichuris trichiura*, hookworm, and *Strongyloides*spp. which are also referred as soil-transmitted helminths (STHs) due to their transmission through soil. STHs infections are widely distributed in tropical and subtropical areas, with most infected people living in developing countries^2^. Poor environmental and personal hygiene and sanitation and contamination of food and drinking water from improper disposal of human excreta are suggested to be major contributors to the high prevalence of STHs^3–5^.

A high prevalence of STHs is widely reported in Nepal, particularly among school-aged children^6–8^. These infections can cause malnutrition and poor growth in children^9^. STHs obtain nutrients needed for survival via the gastrointestinal tract. Associations with malnutrition are possibly mediated through impaired fat digestion, reduced vitamin absorption (particularly vitamin A), and temporary lactose intolerance^10^. Suggested mechanisms for the effects of undernutrition include appetite suppression, increased nutrient loss, and decreased nutrient absorption and utilization^11,12^. Physical damage to the gut is also common, e.g., villus atrophy/hyperplasia, disruption of the mucus barrier and chronic inflammation due to increased gut permeability. Undernutrition is widespread in Nepal; one in three adolescents is stunted (height-for-age z score <-2 SD), one in four boys is undernourished (BMI-for-age z score <- 2 SD), and one in five adolescent girls has anaemia^13^.

According to Engel et al^14^, approximately 200 million children, predominantly from low- and middle-income countries, do not attain their complete cognitive potential, a circumstance deemed in contravention of the child’s rights (United Nations Convention on the Rights of the Child: General Comment No. 7)^14^. A large population-based cohort study in India also reported cognitive deficits among undernourished children^15^. A recent scoping review of 26 articles also indicated the significant impact of being underweight on cognition^16^. Regarding the pathway linking STHs and cognition, epidemiological research has shown that infected school-age children exhibit poorer health outcomes than their uninfected counterparts. These outcomes include a greater incidence of lethargy^17^and anaemia^18,19^. Furthermore, studies have indicated a heightened prevalence of school absenteeism^17^, along with diminished performance across various cognitive assessments^20^. For example, studies have suggested that STHs are associated with poor performance at school^21,22^and low IQ^9^. Considering the likely impacts of STHs on learning ability via morbidity pathways, the WHO recommends monitoring STHs among school-aged children.

Child neglect is not classified as a crime in Nepal, and while severe child abuse is prohibited, it is not categorized as a state offense^23,24^. Additionally, minor instances of physical discipline administered to a child by parents or other family members do not constitute a breach of the law^25^. Conversely, the detrimental impact of exposure to an unfavorable environment has been suggested to have adverse impacts on neurodevelopment^26^. Therefore, the impact of adverse childhood experiences (ACEs) is anticipated to be comparatively more pronounced in Nepal.

We previously reported the adverse effects of *in utero *exposure to toxic elements on neurodevelopment in infants^27^. However, such detrimental effects did not persist at 6, 24 or 36 months of age, while a study showed an association between neurodevelopment and the home environment^28–30^. The effect of STHs on cognition, together with widespread undernutrition and adverse childhood experience (ACE), at a later age was not previously evaluated. Hence, this study aimed to investigate the influence of STHs and nutritional status on the IQ of birth cohort adolescents in the Chitwan Valley, Nepal, while adjusting for ACEs and other sociodemographic characteristics as potential confounders.

## Methods

### Study overview and timeline

From September to October 2008, two hundred pregnant women who decided to deliver at the hospital were approached at Bharatpur General Hospital in the Chitwan district. The detailed eligibility criteria (in brief, living in the study area for at least 2 years, at term pregnancy, age 18–40 years, singleton pregnancy, and no reports of diabetes, hypertension, or preeclampsia) are delineated elsewhere^27,31^. Of the potential participants evaluated for inclusion, 119 met the eligibility criteria, and 100 provided informed consent to participate in the Chitwan Birth Cohort study (84% participation rate)^27^. The study protocol received approval from the ethics committees of the Graduate School of Medicine, University of Tokyo (approval no #2244), and the Bharatpur General Hospital, Chitwan, Nepal.

The birth cohort was tracked after a span of 14 years, utilizing the available addresses of participants’ households and their contact details. The follow-up study protocol received approval from the ethics committees of the National Health Research Council (approval no. NHRC#198–2022) situated in Kathmandu, Nepal, and the University of Tokyo (approval no. 2022260NI-(1)). Between September and October 2022 and 2023, seventy-four participants were visited in their respective homes following prior appointments. The study’s background and objectives were explained to participants and their parents before obtaining informed consent and assent. Informed assent and consent were duly signed by all participants (*n* = 74) and their legal guardians. All procedures conducted in this study with human participants adhered to the principles of the Helsinki Declaration.

## Evaluation of STHs

Participants were instructed to use a clean stick to access a sample of feces about the size of the thumb from the first, middle, and last parts of their stools early in the morning into the given sterile vial, and care was taken not to contaminate the sample with urine or soil. The collected samples were preserved in 2.5% potassium dichromate solution to preserve the parasites and transported to the Central Department of Zoology, Tribhuvan University, for further investigation.

Stool samples were analyzed using the Kato–Katz method, which provides the intensity of infection (an accurate measure of the number of eggs present per gram of stool)^32^. The total number of parasite-stained eggs was counted under a microscope to calculate the number of eggs per gram (epg) in a standard amount of sieved fecal sample. The Kato template was used to extract around 41.7 mg of fecal matter for each smear. To determine the quantitative results, the egg count per smear was multiplied by 24, converting the data into eggs per gram (epg) of fecal material^33^. For quality control, 10% of the smears were randomly selected and evaluated twice. There was no significant difference (paired t test; all *p* > 0.05) between the two evaluations, and the correlation between egg density and age was 0.88.

## Anthropometry of participants

Body weight was accurately recorded to the closest 0.1 kg by employing a portable digital scale (Model BF-046 WH; Tanita, Tokyo, Japan). Height measurements were taken with a precision of 0.1 cm using a stadiometer. The body mass index (BMI) was subsequently computed by dividing the weight (in kilograms) by the square of the height (in square meters). The z scores for BMI (BMIZ) were determined based on age and sex according to the World Health Organization (WHO) growth reference BMI-for-age scores for 5–19 years. Participants were divided into groups of normal weight ( > − 2 SD) and underweight ( < − 2 SD) according to WHO guidelines^34^.

## Interviews with parents and participants during home visits

Structured questionnaires were employed to thoroughly gather a comprehensive array of information through interviews conducted with both parents and participants. This included the current educational attainment of mothers and fathers in terms of years, the annual family income, educational grades, and the most recent grade point average (GPA) of participants. Furthermore, participants were requested to provide self-assessment scores for the quality of their school. Similarly, mothers were asked to provide self-assessment scores for their perceived intelligence and quality of the growth environment. All self-assessments or perceived scores were assigned values ranging from 1 to 5, with 1 representing the lowest and 5 indicating the highest in each category.

## Postnatal growth environment

The authors (RPP and SB) visited the residences of each mother/child pair 14 years after childbirth (with an average time span of 14.04 ± 0.4 years from the baby’s birth). During these visits, the postnatal growth environment was assessed through the application of the Adverse Childhood Experiences Questionnaire (ACE-Q)^35^. The ACE-Q is a 10-item instrument (with potential scores ranging from 0 to 10) designed to quantify occurrences of adverse or traumatic experiences that an individual has encountered prior to reaching the age of 18 years. The ACE-Q assesses an individual’s exposure to various forms of childhood adversity, encompassing psychological, physical, and sexual abuse, along with instances of household dysfunction such as domestic violence, substance misuse, and incarceration^35,36^. The ACE-Q was individually administered by RPP to male participants and by SB to female participants, employing a self-report format to ensure privacy (owing to the potentially triggering nature of some questions related to trauma). Each affirmative response to the questions was tabulated, yielding a cumulative ACE-Q score (ranging from 0 to 10). A higher score signifies a greater number of adverse childhood experiences encountered by the individual, thereby indicating an elevated risk for issues pertaining to social, mental, or general well-being. A score of 4 or more was considered clinically significant^37^.

### Neurodevelopmental indicators such as WASI-II IQ scores

The Wechsler Abbreviated Scale of Intelligence (WASI), introduced in 1999, offers an assessment range spanning from 6 to 89 years. The WASI II comprises four subtests—vocabulary, block design, similarities, and matrix reasoning—requiring approximately 35 min for completion. The WASI-II yields scores for the full-scale intelligence quotient with 4 subsets (FSIQ-4)^38^. The WASI-II INDIA (hereinafter referred to as WASI II), which furnishes a succinct yet dependable assessment of cognitive ability for application in research settings, was selected for this study. The decision to adopt the WASI-II was based on its standardization study conducted on a nationally representative sample encompassing approximately 1,540 individuals aged 6 to 90 years. Notably, this sample is reflective of India’s population and is anticipated to closely correspond to the population and norms of Nepal. The WASI-II is frequently used in the field of neurotoxicology. The WASI-II is considered one of the measures of choice for brief evaluation of intelligence^39^. The psychometric properties, including reliability and validity, have been well-established^39^in prior research using this tool in diverse populations^40,41^.

The WASI II was administered to the children within a span of 4 weeks from the target age group (i.e., 14 years ± 1 month, as stipulated in the WASI II manual), with the age of the children recorded in months and days. The WASI II assessment for all participants was conducted by a skilled researcher (RPP), who maintained a blinded approach to the participants’ exposure status. Rigorous adherence to standardized assessment conditions was upheld, encompassing factors such as providing advanced information about the assessment, minimizing the presence of other individuals, adopting a friendly manner while interacting with the children, ensuring moderate lighting at the assessment location, and considering the participants’ well-being (e.g., absence of hunger or sleepiness). The assessment of participants was conducted within the familiar confines of their own homes, chosen for convenience for both the caregivers and the cohort children (thus avoiding any potential hesitancy due to a less familiar setting).

## Translation and training

All tests and interviews were meticulously translated (and subsequently back-translated) between Nepali and English while ensuring the inclusion of culturally appropriate adjustments. The Research Assistant (coauthor SB) underwent comprehensive training (conducted by Principal Investigator RPP) and engaged in supervised practice sessions spanning a period of two weeks. Prior to analysis, a thorough review was conducted to ascertain the validity of the written test responses, encompassing assessments of adherence to acceptable ranges and the presence of any missing data prior to data entry.

### Statistical analysis

First, the normality of the distribution of all the variables was examined and skewed variables were log-transformed.

Differences in the sociodemographic, socioeconomic, growth environmental and rated characteristics were investigated using the independent t test for continuous variables, by infection status or by IQ cutoff status (Tables 1 and 3). Similarly, differences in behavioral and lifestyle characteristics were examined by Fisher’s exact test or the chi-square test, which are used to analyze categorical variables (Tables 2 and 3). Sociodemographic, socioeconomic, behavioral and lifestyle characteristics were examined for associations with STH incidence using univariate and multivariate logistic regression models for mutual adjustment (Table 4). Similarly, STHs prevalence, and nutritional status were examined for associations with developmental delay (IQ < 69) using univariate and multivariate logistic regression models for mutual adjustment (Table 3).

Bivariate (Model 1) and multivariate (Model 2) analyses were also conducted to examine the (unadjusted and fully adjusted associations, respectively) associations between WASI II FSIQ scores and the presence of *A. lumbricoides*^9,42,43^, undernutrition^15,16^, maternal education^41,44–46^, annual family income^22,47–49^and the ACE-Q score^50,51,]^adjusting for participant sex and age at the time of the WASI II assessment^52^, which are known to correlate with neurodevelopmental indicators such as IQ (Table 4).

In Model 3, the explanatory variables (presence and egg density of *A. lumbricoides* infections) were adjusted solely for the “significantly associated covariates” identified (i.e., z scores for BMI, maternal education and log annual income) from the univariate model (Model 1). This adjustment aimed to ascertain the “minimal” impact of explanatory variables and covariates on the response variables. Model 2 involved the implementation of a multivariate regression model, thereby evaluating the comprehensive (i.e., full) adjusted influence of the explanatory variables and covariates on the response variables.

Among the 100 initially recruited mother–infant pairs within the cohort, follow-up data spanning 14 years were available for 74 participants. A p value less than 0.05 indicated statistical significance. The statistical analyses were performed utilizing SPSS version 25 (SPSS, IBM Corporation).

## Results

Table 1 summarizes the characteristics of the mother-adolescent pairs at the 14-year follow-up. None of the characteristics differed significantly according to the status of parasitic infection. Individuals with an extremely low IQ indicating delayed development (i.e., FSIQ < 69) were considered to have a low height, inferior nourishment, low maternal education, and low annual family income, and their mother had an inferior growth environment score compared to that of the children with an FSIQ > 69. As anticipated, participants with extremely low IQs (i.e., < 69) exhibited low GPA and low mother-rated academic performance. There was no significant difference between infected and uninfected participants in nutritional status (i.e., BMI categories) or behavioral practices. The majority of participants did not trim their nails regularly, did not consume anthelmintics within the past 6 months, played in soil, and did not drink boiled or filtered water. Positive health behaviors (i.e., handwashing with soap before eating) were more frequently observed among uninfected participants (Supplementary Table 1).

**Table 1:** Characteristics of study participants (*n* = 74).

Characteristics	Not-infected with STHs (*n* = 62)	Infected with STHs (*n* = 12)	t test *p* value^$^	FSIQ > Cut off of 69^ (*n* = 49)	FSIQ < Cut off of 69 (*n* = 25)	t test *p* value^$^	Total
	Mean (SD)	Mean (SD)		Mean (SD)	Mean (SD)		Mean (SD)
**Demographic and nutritional Characteristics**							
Age (years)	14.04 (0.04)	14.04 (0.04)	NS	14.04 (0.04)	14.04 (0.04)	NS	14.04 (0.04)
Height (Meter)	1.58 (0.09)	1.58 (0.06)	NS	**1.59 (0.08)**	**1.55 (0.08)**	**0.019**	1.58 (0.08)
Weight (kg)	46.61 (9.99)	46.08 (8.46)	NS	48.38 (9.06)	42.74 (10.08)	NS	46.52 (9.71)
Body Mass Index (BMI)(kg/m2)	18.59 (3.25)	18.51 (3.03)	NS	19.04 (2.88)	17.64 (3.63)	NS	18.58 (3.19)
BMI for Z Score	-0.61 (1.42)	-0.64 (1.62)	NS	**-0.34 (1.23)**	**-1.16 (1.69)**	**0.01**	-0.61 (1.44)
**Socioeconomic characteristics**							
Maternal years of education (years)	9.40 (4.11)	9.55 (4.34)	NS	**10.46 (3.86)**	**7.44 (3.94)**	**0.002**	9.42 (4.12)
Annual family income (1,00,000 NPR)	6.19 (4.82)	7.60 (7.95)	NS	**7.73 (6.12)**	**3.74 (1.55)**	**< 0.001**	6.42 (5.42)
**Growth environment characteristics**							
Adverse Childhood Environment (ACE)	0.76 (1.34)	0.42 (0.51)	NS	0.69 (1.25)	0.72 (1.28)	NS	0.70 (1.25)
Mother rated growth environment (1–5)	4.44 (0.88)	4.42 (0.67)	NS	**4.63 (0.60)**	**4.08 (1.12)**	**0.03**	4.44 (0.85)
Number of people in same residence	4.73 (1.48)	4.75 (2.67)	NS	4.86 (2.00)	4.48 (0.87)	NS	4.73 (1.71)
Self-rated quality of school (1–5)	4.00 (0.92)	3.55 (0.82)	NS	3.89 (0.95)	4.02 (0.83)	NS	3.93 (0.91)
Self-reported screen time per days (hours)	2.61 (2.67)	2.22 (1.74)	NS	2.56 (2.34)	2.52 (1.87)	NS	2.55 (2.19)
How many days you lost school (days)	7.10 (8.24)	6.39 (4.86)	NS	6.73 (7.60)	7.45 (8.87)	NS	6.99 (7.78)
**Mother**,** school and scale rated characteristics**			NS				
Mother rated intelligence of their child (1–5)	4.16 (0.81)	4.25 (0.75)	NS	4.20 (0.79)	4.12 (0.83)	NS	4.18 (0.80)
Mother rated academic performance (1–5)	3.66 (0.98)	3.46 (0.89)	NS	**3.99 (0.73)**	**2.92 (0.99)**	**< 0.001**	3.63 (0.96)
Reported Grade Point Average (GPA)	3.05 (0.62)	3.12 (0.39)	NS	**3.25 (0.39)**	**2.59 (0.73)**	**0.01**	3.06 (0.59)
Full Scale intelligence quotient (FSIQ)	74.44 (15.15)	75.58 (16.59)	NS	-	-	-	74.62 (15.28)

^$^ Independent t test, NS: not significant, NRS: Nepali rupee, STHs: soil-transmitted helminths, FSIQ: full-scale intelligence quotient.

^FSIQ < 69 is considered an extremely low IQ indicating developmental delay (Wechsler 2011).

Table 2 shows the prevalence of STHs among the study participants. Only 12 samples (16.2%) demonstrated shedding of eggs from *A. lumbricoides*. There was a greater prevalence of *A. lumbricoides* in male participants than in female participants. Egg density (epg) was greater among females than among males, but this difference was not statistically significant. Out of the 74 cohort participants followed up, 25—which was more than one-third (i.e., 33.78%) of participants—indicated an extremely low intelligence quotient (IQ) evaluated by the Wechsler Abbreviated Scale of Intelligence (WASI). There was no difference in roundworm incidence or egg density when participants were compared based on neurodevelopmental performance (i.e., IQ < 69 vs. > 70, the cut-off for delayed development). The prevalence of *A. lumbricoides* was not associated with any of the behavioral characteristics evaluated. However, participants who reported not using soap to wash their hands before eating, participants who reported a nail-biting habit, and participants who reported playing in soil had increased odds of contracting ascariasis compared to their counterparts. Again, none of the evaluated associations reached statistical significance (Supplementary Table 2).

**Table 2:** Prevalence and egg density of *A. lumbricoides* in Chitwan birth cohort participants (*n* = 74).

Parasite species *A. lumbricoides*	Male *n* (%)	Female *n* (%)	*p*- value	FSIQ > Cut off of 69 (*n* = 49) *n* (%)	FSIQ < Cut off of 69^ (*n* = 25) *n* (%)	*p* value	Total
STHs Prevalence	7 (20.6)	5 (12.5)	NS*	9 (18.4)	3 (12.0)	NS^#^	12 (16.2)
**Infection intensity**							
	**mean (SD)**	**mean (SD)**		**mean (SD)**	**mean (SD)**		**mean (SD)**
Egg per gram (epg)	8.47 (17.61)	16.20 (57.62)	NS^$^	16.16 (52.40)	5.76 (17.36)	NS^$^	12.65 (43.92)

^$^ Independent t test, * Chi-square test, ^#^ Fisher exact test, NS: not significant, SD: standard deviation, STHs: soil-transmitted helminths, FSIQ: full-scale intelligence quotient, ^FSIQ < 69 is considered an extremely low IQ indicating developmental delay (Wechsler 2011).

Table 3 shows the associations of *A. lumbricoides* infection and nutritional status with developmental delay. The prevalence of *A. lumbricoides* and sex were not associated with neurodevelopmental delay according to either the univariate or multivariate models. Participants with BMIZ <-2SD (i.e., underweight) had higher odds ratios of neurodevelopmental delays than did their counterparts according to both the univariate and multivariate models.

**Table 3:** Odds of developmental delay with respect to STHs infection and nutritional status (*n* = 74).

	FSIQ < 69^ (*n* = 74)		
	Univariate		Multivariate^#^
	n (%)	OR (95%CI)	AOR (95%CI)
**Contextual factors**			
*A lumbricoides* prevalence			
No	88.0	ref	ref
Yes	12.0	0.61 (0.15 to 2.47)	0.58 (0.12 to 2.72)
BMIZ Category			
BMIZ > -2SD (Normal)	24.6	**ref**	**ref**
BMIZ <-2SD (Underweight)	75.0	**9.20 (2.20 to 38.47)**	**9.41 (2.23 to 39.80)**

OR: odds ratio, AOR: adjusted odds ratio, 95% CI: 95% confidence interval, %: prevalence percentage, ref: reference. BMIZ: z scores for body mass index (BMI); FSIQ: Full Scale Intelligence Quotient; ^FSIQ < 69 is considered an extremely low IQ indicating developmental delay (Wechsler 2011). ^#^ In the multivariate model, STHs infection and BMIZ category were mutually adjusted.

Table 4 summarizes the associations between *A. lumbricoides* egg density and WASI-II full-scale intelligence quotient (FSIQ) scores. The FSIQ scores were normally distributed (Supplementary Fig. 1). None of the associations (Model 1: bivariate; Model 2: fully adjusted; Model 3: minimally adjusted multivariate model) achieved statistical significance (i.e., *p* < 0.05). Among the contextual covariates, mother’s education level and reported annual family income were significantly (*p* < 0.05) positively associated with IQ scores, with consistent significance and direction in all 3 models.

**Table 4:** Association of *A. lumbricoides* egg density, nutritional and contextual variables with FSIQ scores of WASI II at 14 years (*n* = 74).

Response Variables	FSIQ score		
**Explanatory variables**	Model 1^a^	Model 2 ^b^	Model 3^c^
**Predictor Variables**	Estimated difference (95% confidence interval) in the value of the response variable for a one unit increase of the explanatory variable		
*Ascaris lumbricoides* egg density (epg)	–0.00 (–0.08 to 0.08)	–0.02 (–0.08 to 0.05)	–0.02 (–0.08 to 0.05)
**Covariates**			
**Participant’s Parent’s characteristics**			
Mother’s Education level (years)	**1.94 (1.19 to 2.70)**	**1.44 (0.65 to 2.23)**	**1.34 (0.59 to 2.08)**
**Participants characteristics**			
BMIZ Score	**2.50 (0.06 to 4.94)**	1.22 (-0.81 to 3.25)	1.23 (-0.80 to 3.25)
**Growth Environment characteristics**			
Adverse childhood experience (ACE) total score	–1.02 (–3.89 to 1.85)	0.96 (–1.46 to 3.38)	-
Log annual Income (1,00,000 NRS)	**29.41 (18.85 to 39.96)**	**20.37 (9.36 to 31.37)**	**20.60 (9.65 to 31.56)**
R^2^		**0.433**	**0.428**

WASI: Wechsler Abbreviated Scale of Intelligence, FSIQ: Full-scale Intelligence Quotient, NRS: Nepali rupees, R^2^: R-square, epg: egg per gram of face, BMIZ: z score for body mass index (BMI), WASI II: Wechsler Abbreviated Scale of Intelligence. Model 1^a^: A bivariate regression assessed the unadjusted associations between *A. lumbricoides* egg density and covariates (maternal education, ACE score, log annual income). Model 2^b^: A multivariate regression analyzed the adjusted effects of the explanatory and covariates on FSIQ. Model 3^c^: *A. lumbricoides* egg density was adjusted for only the significant covariates (maternal education, log income) from Model 1 to estimate the minimum adjusted effect on FSIQ.

## Discussion

Neither the presence nor the egg density of *A. lumbricoides* infections was associated with the cognitive performance (WASI IQ) score of the cohort participants according to univariate or multivariate models. Associations remained insignificant in both analyses [i.e., categorical according to logistic regression and continuous variable evaluation via linear regression]. However, *A. lumbricoides*egg density (epg) exhibited a nonsignificant but consistent inverse association in both the univariate and multivariate models (Models 2 and 3) after adjustment for these variables (Table 4). Consistent insignificant findings were reported among primary school pupils in Ile-Ife, Osun State, Nigeria^53,]^ with a comparable prevalence (22.1%) of *A. lumbricoides* but almost double the percentage of participants (65.4%) who experienced cognitive deficits. In contrast, the presence of *A. lumbricoides*infections was associated with significantly lower cognitive scores in school-age children in Java, Indonesia^43^. Improved cognitive function in primary schoolchildren infected with *A. lumbricoides*was reported after five months of receiving this intervention in the northern region of Jakarta, Indonesia^42^. In addition, pooled results from a systematic review and meta-analysis of 36 observational and intervention studies indicated the cognitive benefit of deworming for helminth infections^9^. Out of 25 participants who indicated delayed development (IQ < 69), only 3 (12%) participants reported *A. lumbricoides* infections. Hence, these nonsignificant associations may also be due to the small sample size, which resulted in a lack of statistical power.

This study also revealed that undernutrition (BMIZ<–2SD) increased the odds of a developmental deficit according to both the univariate and the adjusted multivariate models. The negative impact of underweight status on the cognitive neurodevelopment of children is well documented. For example, a scoping review of 26 articles indicated a significant impact of underweight on cognitive neurodevelopment^16^. A recent population-based cohort study in India with a large sample size (*n*= 41,554) also reported cognitive deficits among undernourished children^15^. However, the lack of an association between BMI and IQ according to linear regression models urges careful interpretation of the association. Yet, *Ascariasis *may lead to issues such as impaired fat digestion, reduced vitamin absorption, and temporary lactose intolerance^10^. These problems can result in decreased appetite, increased nutrient loss, and reduced nutrient uptake^11,12^, collectively contributing to low BMIZ. When BMIZ falls below a certain threshold (i.e., <-2SD), individuals who are underweight may experience weakness or lethargy^17^due to anemia^18,19^or severe weight loss, which can lead to higher rates of school absenteeism^17^and cognitive deficits^9^. This illustrates a complex relationship between ascariasis, malnutrition and cognitive decline. The scenario of chance finding cannot be discounted.

This study also investigated the prevalence of STHs among Chitwan birth cohort participants followed up after 14 years of birth. The prevalence of *A. lumbricoides*in our study (i.e., 16.2%) was slightly lower than the prevalence reported in various studies in Nepal, such as 20% among schoolchildren in Kathmandu, Nepal^54^; 21.8% among public and private schoolchildren in Kathmandu, Nepal^55^; and 22.63% among schoolchildren in the Bhaktapur district, Nepal^56^. A lower prevalence of *A. lumbricoides* was also recently reported among school-age adolescents—8.33% among primary school children in Bhaktapur, Nepal^57^; 5.5% among private school-going pupils in the Dharan Sub metropolitan City, Nepal^58^; 2.3% among rural area school children in the Lokhim VDC, Nepal^59^; and 1.8% among school children in the Saptari District, Nepal^60^. In contrast, a high prevalence of *A. lumbricoides *has also been reported in many previous studies. For example, a 50.92% prevalence of ascariasis was reported in schoolchildren in the Rangeli Municipality of Morang District in Eastern Nepal^6^. Similarly, a recent study among schoolchildren in Kritipur Municipality, Kathmandu, reported a 34.2% prevalence of *A. lumbricoides*^8^. Another study among children from five schools in Bhairahawa, Nepal, reported a 29.1% overall prevalence of *A. lumbricoides*^7^. This discrepancy in the prevalence of STHs might be due to differences in climatic conditions and microecology^61^, population characteristics, and awareness of steps of prevention^62^.

Despite coverage of the study area by the national deworming campaign in Nepal since 2004^63^, the 16.2% prevalence of *A. lumbricoides* in the study area is unexpected and relatively high. This may be partially explained by prevailing unhygienic behaviors and lifestyles (Supplementary Table 1) among participants. For example, most of the participants (i.e., 90%) reported that they did not consume anthelmintic drugs that are distributed freely in schools. In addition, more than two-thirds of the participants (67.6%) did not use soap to wash their hands before eating food by hand; 92% of the participants reported inadequate hand hygiene behavior and presented with ascariasis infections. Furthermore, approximately two-thirds of the participants reported that they played in soil but did not trim their nails regularly. In addition, the high prevalence of *A. lumbricoides* infection may also be attributed to the extremely resistant nature of Ascaris eggs, which contributes significantly to transmission dynamics. However, the small sample size limits our ability to generalize the findings.

The prevalence and egg density of *A. lumbricoides* were similar between males and females and did not differ significantly based on IQ status. None of the behavioral, lifestyle, socioeconomic, or demographic factors were associated with the prevalence of *A. lumbricoides*. Even hand-washing habits, which differed significantly by infection status, lost statistical significance in logistic regression according to both the univariate and multivariate models. Limitations of the small sample size might be behind this inconsistency due to the lack of statistical power. Hence, future studies with larger sample sizes need to be conducted to confirm these associations.

As anticipated, maternal education and reported annual income were consistently significantly associated with participants’ cognitive performance according to both univariate and multivariate models. Mothers’ education is an established contributor to their children’s IQ^41,44–46^. This study also confirmed the positive association with full-scale IQ. Although a positive association between reported annual income and IQ has been suggested by many other studies^22,47–49^, the direction of the association and the large effect size, even after full adjustment for other variables, such as maternal education, BMIZ, ACE, and other relevant postnatal environment variables, are noteworthy. Participants with educated mothers and greater annual family income may have a more enriched environment, which might ultimately contribute to better IQ scores. Social determinants of health are nonmedical social and environmental factors such as level of education, income, relationships, housing conditions, and social status that impact an individual’s health status. Social determinants contribute to health inequities, as individuals who consistently experience sociodemographic disadvantages demonstrate worse health outcomes than their counterparts. The WHO estimates that factors considered to be social determinants may account for 30–55% of health outcomes^64^.

A few limitations of this study should be considered. The first and main methodological limitation of the study is the use of single Kato-Katz smear assessments, which may be inadequate for fully assessing long-term parasitic burden and can be sensitive to day-to-day and within-stool variations in eggs per gram (epg) of stool. However, the Kato–Katz method is the method of choice for measuring the number of eggs present^32^. Second, the limited sample size, especially in subgroup analyses, could have heightened the risk of type II error, reducing the likelihood of identifying more subtle associations. Consequently, the study’s statistical power might be inadequate for detecting certain effects. Moreover, the generalizability of the findings could be constrained, as the sample may not accurately reflect the wider population. Further studies with larger and more diverse groups are necessary to validate and expand upon these results. Third, while IQ was assessed using the WASI II by a trained researcher, and key measures such as the ACE score were also conducted by research staff, some subjective assessments provided by parents or participants (e.g., growth environment, school quality, and academic performance) may not fully align with objective measures. Additionally, no interim health assessments, such as diarrheal episodes or breastfeeding practices, were conducted, which may have influenced cognitive outcomes during early childhood.

## Conclusion

Despite the coverage of an ongoing national deworming program since 2004 in the study area, this cohort of participants indicated a relatively high prevalence (i.e., 16%, (12/74)) of *A. lumbricoides*. Developmental deficits among one-third of participants might have stemmed from their morbidity associated with undernutrition (16%, (12/73)). Although none of the lifestyle, hygiene or behavioral factors explained the increase (infection of *A. lumbricoides)*,* the* high prevalence of unhealthy lifestyles and inadequate hygiene behaviors highlights the need for, and importance of, “in-depth” health and nutritional education.

## Electronic supplementary material

Below is the link to the electronic supplementary material.

Supplementary Material 1

## Data Availability

The datasets used during the current study are available from the corresponding author upon reasonable request.

## References

[1] World Health Organization. Soil-transmitted helminth infections: key facts; updated 2 march 2020. Available online(accessed 11 June. (2020). (2021).

[2] Chan, M. S., Medley, G. F., Jamison, D. & Bundy, D. A. P. The evaluation of potential global morbidity attributable to intestinal nematode infections. *Parasitology*. **109**, 373–387 (1994). 7970892 10.1017/s0031182000078410

[3] Feleke, D. G., Alemu, Y., Bisetegn, H., Mekonnen, M. & Yemanebrhane, N. Intestinal parasitic infections and associated factors among street dwellers and prison inmates: a systematic review and meta-analysis. *PLoS One*. **16**, e0255641 (2021). 34352000 10.1371/journal.pone.0255641 PMC8341648

[4] Boonjaraspinyo, S. et al. Prevalence and Associated Risk factors of intestinal parasitic infections: a Population-based study in Phra Lap Sub-district, Mueang Khon Kaen District, Khon Kaen Province, Northeastern Thailand. *Trop. Med. Infect. Dis. ***8**, (2022). 10.3390/tropicalmed8010022 PMC9860576 36668929

[5] Mosisa, G. et al. Burden of intestinal parasitic infections and associated factors among pregnant women in East Africa: a systematic review and meta-analysis. *Matern Heal Neonatol Perinatol. ***9**, 5 (2023). 10.1186/s40748-023-00150-8 PMC10077618 37020236

[6] Yadav, S. N. & Mahato, S. Study on intestinal helminth parasites in school children of Rangeli Municipality of Morang District in Eastern Nepal. *Am. J. Heal Res. ***5**, 50–53 (2017).

[7] Raut, S., Bhattarai, S., Khanal, R., Khatiwada, S. & Kasarla, R. R. Burden of intestinal parasitic infections among children from five schools in Bhairahawa, Nepal: a comparative cross-sectional study. *J. Univers. Coll. Med. Sci. ***9**, 30–35 (2021).

[8] Dahal, A., Roka, D. B., Prasad, S. M. & Shrestha, S. Prevalence of intestinal parasitic infection among school children in Kirtipur Municipality, Kathmandu. *Nepal. Med. Coll. J. ***24**, 121–126 (2022).

[9] Pabalan, N. et al. Soil-transmitted helminth infection, loss of education and cognitive impairment in school-aged children: a systematic review and meta-analysis. *PLoS Negl. Trop. Dis. ***12**, e0005523 (2018). 29329288 10.1371/journal.pntd.0005523 PMC5766095

[10] World Health Organization. *Prevention and Control of Schistosomiasis and Soil-Transmitted Helminthiasis: Report of a WHO Expert Committee* (World Health Organization, 2002). 12592987

[11] Stephenson, L. S., Latham, M. C. & Ottesen, E. A. Malnutrition and parasitic helminth infections. *Parasitology*. **121**, S23–S38 (2000). 11386688 10.1017/s0031182000006491

[12] De Silva, N. R. Impact of mass chemotherapy on the morbidity due to soil-transmitted nematodes. *Acta Trop. ***86**, 197–214 (2003). 12745137 10.1016/s0001-706x(03)00035-4

[13] Ministry of Health and Population (MOHP). Nepal National Micro-nutrient Status Survey (NNMSS), 2016. *Kathmandu, Nepal *(2018).

[14] Engle, P. L. et al. Strategies for reducing inequalities and improving developmental outcomes for young children in low-income and middle-income countries. *Lancet*. **378**, 1339–1353 (2011). 21944378 10.1016/S0140-6736(11)60889-1

[15] Soni, A. et al. Early childhood undernutrition, preadolescent physical growth, and cognitive achievement in India: a population-based cohort study. *PLoS Med. ***18**, e1003838 (2021). 34705825 10.1371/journal.pmed.1003838 PMC8580255

[16] Suryawan, A. et al. Malnutrition in early life and its neurodevelopmental and cognitive consequences: a scoping review. *Nutr. Res. Rev. ***35**, 136–149 (2022). 34100353 10.1017/S0954422421000159

[17] Nokes, C. et al. Moderate to heavy infections of Trichuris Trichiura affect cognitive function in Jamaican school children. *Parasitology*. **104**, 539–547 (1992). 1641252 10.1017/s0031182000063800

[18] Nokes, C. & Bundy, D. A. P. Compliance and absenteeism in school children: implications for helminth control. *Trans. R Soc. Trop. Med. Hyg. ***87**, 148–152 (1993). 8337713 10.1016/0035-9203(93)90464-2

[19] Grantham-McGregor, S. Linear growth retardation and cognition. *Lancet*. **359**, 542 (2002). 11867104 10.1016/S0140-6736(02)07719-X

[20] Savioli, L., Bundy, D. & Tomkins, A. Intestinal parasitic infections: a soluble public health problem. *Trans. R Soc. Trop. Med. Hyg. ***86**, 353–354 (1992). 1440799 10.1016/0035-9203(92)90215-x

[21] Guan, M. & Han, B. Association between intestinal worm infection and malnutrition among rural children aged 9–11 years old in Guizhou Province, China. *BMC Public. Health*. **19**, 1–11 (2019). 31477069 10.1186/s12889-019-7538-y PMC6719348

[22] Akubuilo, U. C. et al. Academic performance and intelligence quotient of primary school children in Enugu. *Pan Afr. Med. J. ***36**, (2020). 10.11604/pamj.2020.36.129.22901 PMC7422740 32849984

[23] Kandel, P., Kunwar, R., Karki, S., Kandel, D. & Lamichhane, P. Child maltreatment in Nepal: prevalence and associated factors. *Public. Health*. **151**, 106–113 (2017). 28763786 10.1016/j.puhe.2017.06.020

[24] Neupane, D. et al. Self-reported child abuse in the home: a cross-sectional survey of prevalence, perpetrator characteristics and correlates among public secondary school students in Kathmandu, Nepal. *BMJ Open. ***8**, e018922 (2018). 29921678 10.1136/bmjopen-2017-018922 PMC6009519

[25] Elijah Davis. Representing Children Worldwide: Nepal. *Yale University* (2016). https://rcw.law.yale.edu/jurisdiction-research/nepal

[26] Anda, R. F. et al. The enduring effects of abuse and related adverse experiences in childhood: a convergence of evidence from neurobiology and epidemiology. *Eur. Arch. Psychiatry Clin. Neurosci. ***256**, 174–186 (2006). 16311898 10.1007/s00406-005-0624-4 PMC3232061

[27] Parajuli, R. P., Fujiwara, T., Umezaki, M. & Watanabe, C. Association of cord blood levels of lead, arsenic, and zinc with neurodevelopmental indicators in newborns: a birth cohort study in Chitwan Valley, Nepal. *Environ. Res. ***121**, 45–51 (2013). 23164520 10.1016/j.envres.2012.10.010

[28] Parajuli, R. P., Fujiwara, T., Umezaki, M. & Watanabe, C. Home environment and cord blood levels of lead, arsenic, and zinc on neurodevelopment of 24 months children living in Chitwan Valley, Nepal. *J. Trace Elem. Med. Biol. ***29**, 315–320 (2015). 25213681 10.1016/j.jtemb.2014.08.006

[29] Parajuli, R. P., Fujiwara, T., Umezaki, M., Furusawa, H. & Watanabe, C. Home environment and prenatal exposure to lead, arsenic and zinc on the neurodevelopment of six-month-old infants living in Chitwan Valley, Nepal. *Neurotoxicol Teratol*. **41**, 89–95 (2014). 24418190 10.1016/j.ntt.2013.12.006

[30] Parajuli, R. P., Umezaki, M., Fujiwara, T. & Watanabe, C. Association of cord blood levels of lead, arsenic, and zinc and home environment with children neurodevelopment at 36 months living in Chitwan Valley, Nepal. *PLoS One*. **10**, e0120992 (2015). 25803364 10.1371/journal.pone.0120992 PMC4372553

[31] Parajuli, R. P. et al. Cord blood levels of toxic and essential trace elements and their determinants in the Terai region of Nepal: a birth cohort study. *Biol. Trace Elem. Res. ***147**, 75–83 (2012). 22234823 10.1007/s12011-011-9309-1

[32] Montresor, A. et al. Guidelines for the Evaluation of Soil-Transmitted Helminthiasis and Schistosomiasis at Community Level: A Guide for Managers of Control Programmes. (1998).

[33] Swiss, T. & Institute, B. KATO-Katz technique for helminth eggs. *Methods in Parasitology* 2–8 (2005). https://www.iacld.com/UpFiles/Documents/291ecc62-4080-4a22-ab44-9d7b836ce511.pdf

[34] World Health Organization. Growth Reference Data for 5–19 Years. (2007).

[35] Felitti, V. J. et al. Relationship of childhood abuse and household dysfunction to many of the leading causes of death in adults. The adverse childhood experiences (ACE) study. *Am. J. Prev. Med. ***14**, 245–258 (1998). 9635069 10.1016/s0749-3797(98)00017-8

[36] Webster, E. M. The impact of adverse childhood experiences on health and development in young children. *Glob Pediatr. Heal*. **9**, 2333794X221078708 (2022). 10.1177/2333794X221078708 PMC8882933 35237713

[37] Hughes, K. et al. The effect of multiple adverse childhood experiences on health: a systematic review and meta-analysis. *Lancet Public. Heal*. **2**, e356–e366 (2017). 10.1016/S2468-2667(17)30118-4 29253477

[38] Wechsler, D. *Wechsler Abbreviated Scale of Intelligence- Second Edition (WASI-II)* (NCS Pearson INC., 2011).

[39] Mccrimmon, A. & Smith, A. Review of the Wechsler Abbreviated Scale of Intelligence, Second Edition (WASI-II). *J. Psychoeduc Assess. ***31**, 337–341 (2012).

[40] Fatma, R., Chauhan, W., Shahi, M. H. & Afzal, M. Association of BDNF gene missense polymorphism rs6265 (Val66Met) with three quantitative traits, namely, intelligence quotient, body mass index, and blood pressure: a genetic association analysis from North India. *Front. Neurol. ***13**, 1035885 (2022). 36742047 10.3389/fneur.2022.1035885 PMC9894895

[41] Hossain, S. J., Tofail, F., Sujan, H. M., Arifeen, S. E. & Hamadani, J. Factors associated with school achievement of children aged 8–10 years in rural Bangladesh: findings from a post hoc analysis of a community-based study. *PLoS One*. **16**, e0254693 (2021). 34320021 10.1371/journal.pone.0254693 PMC8318268

[42] Hadidjaja, P. et al. The effect of intervention methods on nutritional status and cognitive function of primary school children infected with Ascaris lumbricoides. *Am. J. Trop. Med. Hyg. ***59**, 791–795 (1998). 9840600 10.4269/ajtmh.1998.59.791

[43] Sakti, H. et al. Evidence for an association between hookworm infection and cognitive function in Indonesian school children. *Trop. Med. Int. Health*. **4**, 322–334 (1999). 10402967 10.1046/j.1365-3156.1999.00410.x

[44] Ye, A. et al. Maternal intelligence quotient and motor development in early childhood: the mediating role of mother’s education. *J. Paediatr. Child. Health*. **55**, 87–94 (2019). 30051946 10.1111/jpc.14123

[45] Guo, J. et al. Prenatal exposure to mixture of heavy metals, pesticides and phenols and IQ in children at 7 years of age: the SMBCS study. *Environ. Int. ***139**, 105692 (2020). 32251899 10.1016/j.envint.2020.105692

[46] Sentenac, M. et al. Maternal education and cognitive development in 15 European very-preterm birth cohorts from the RECAP Preterm platform. *Int. J. Epidemiol. ***50**, 1824–1839 (2021). 34999864 10.1093/ije/dyab170

[47] Black, M. M., Hess, C. R. & Berenson-Howard, J. Toddlers from low-income families have below normal mental, motor, and behavior scores on the revised Bayley scales. *J. Appl. Dev. Psychol. ***21**, 655–666 (2000).

[48] Trude, A. C. B. et al. Effects of responsive caregiving and learning opportunities during pre-school ages on the association of early adversities and adolescent human capital: an analysis of birth cohorts in two middle-income countries. *Lancet Child. Adolesc. Heal*. **5**, 37–46 (2021). 10.1016/S2352-4642(20)30309-6 PMC7763480 33340466

[49] Tian, J. et al. Associations between life-course household wealth mobility and adolescent physical growth, cognitive development and emotional and behavioral problems: a birth cohort in rural western China. *Front. Public. Heal*. **11**, 1061251 (2023). 10.3389/fpubh.2023.1061251 PMC9934056 36817901

[50] Sideli, L. et al. Childhood maltreatment, educational attainment, and IQ: findings from a multicentric case-control study of first-episode psychosis (EU-GEI). *Schizophr Bull. ***48**, 575–589 (2022). 35137235 10.1093/schbul/sbac004 PMC9077421

[51] Tognin, S. et al. Impact of adverse childhood experiences on educational achievements in young people at clinical high risk of developing psychosis. *Eur. Psychiatry*. **66**, e16 (2023). 36649929 10.1192/j.eurpsy.2022.2351 PMC9970149

[52] Nwatah, V. E., Ahmed, P. A., Audu, L. I. & Okolo, S. N. Socio-demographic factors influencing measures of cognitive function of early adolescent students in abuja, Nigeria. *Niger Postgrad. Med. J. ***29**, 317–324 (2022). 36308261 10.4103/npmj.npmj_157_22

[53] Olopade, B. O., Idowu, C. O., Oyelese, A. O. & Aboderin, A. O. Intestinal parasites, nutritional status and cognitive function among primary School pupils in Ile-Ife, Osun State, Nigeria. *Afr. J. Infect. Dis. ***12**, 21–28 (2018). 30109282 10.21010/ajid.v12i2.4 PMC6085742

[54] Khanal, L. K. et al. Prevalence of intestinal worm infestations among school children in Kathmandu, Nepal. *Nepal. Med. Coll. J. ***13**, 272–274 (2011). 23016478

[55] Shrestha, J., Bhattachan, B., Rai, G., Park, E. Y. & Rai, S. K. Intestinal parasitic infections among public and private schoolchildren of Kathmandu, Nepal: prevalence and associated risk factors. *BMC Res. Notes*. **12**, 192 (2019). 30925938 10.1186/s13104-019-4225-0 PMC6441203

[56] Shrestha, R. & Maharjan, M. Prevalence of intestinal helminth parasites among school-children of Bhaktapur district, Nepal. *Nepal. J. Zool. ***1**, 48–58 (2013).

[57] Sukupayo, P. & Karmacharya, A. Prevalence of gastrointestinal parasites among primary school children in Bhaktapur, Nepal. *Nepal. J. Zool. ***7**, 20–25 (2023).

[58] Shrestha, B. K. et al. Prevalence and Related Risk Factors of Intestinal Parasitosis among Private School-Going Pupils of Dharan Submetropolitan City, Nepal. *J. Parasitol. Res.* 6632469 (2021). (2021). 10.1155/2021/6632469 PMC8285192 34306741

[59] Rai, L., Saud, B., Paudel, G. & Dhungana, G. Prevalence of intestinal parasitic infection among rural area school children of Lokhim VDC, Nepal. *J. Microbiol. Exp. ***4**, 102 (2017).

[60] Gupta, R. et al. Prevalence of intestinal parasitosis and associated risk factors among school children of Saptari district, Nepal: a cross-sectional study. *Trop. Med. Health*. **48**, 1–9 (2020). 32848503 10.1186/s41182-020-00261-4 PMC7444033

[61] Parajuli, R. P., Umezaki, M. & Watanabe, C. Behavioral and nutritional factors and geohelminth infection among two ethnic groups in the Terai region, Nepal. *Am. J. Hum. Biol. Off J. Hum. Biol. Assoc. ***21**, 98–104 (2009). 10.1002/ajhb.20825 18802944

[62] Adhikari, R. B., Parajuli, R. P., Maharjan, M. & Ghimire, T. R. Prevalence and risk factors for gastrointestinal parasites in the chepangs in Nepal. *Ann. Parasitol. ***67**, 387–405 (2021). 34953115 10.17420/ap6703.353

[63] Kunwar, R., Acharya, L. & Karki, S. Decreasing prevalence of intestinal parasitic infections among school-aged children in Nepal: a systematic review and meta-analysis. *Trans. R Soc. Trop. Med. Hyg. ***110**, 324–332 (2016). 27268711 10.1093/trstmh/trw033

[64] World Health Organization. Social determinants of health. (2024). https://www.who.int/health-topics/social-determinants-of-health#tab=tab_1

