# Supplementary material for: Risk factors for Ascaris lumbricoides infection and its association with nutritional status and IQ in 14-Year old adolescents in Chitwan, Nepal
Source: Sci Rep. 2024 Oct 29;14:26005. doi: 10.1038/s41598-024-77306-9 (PMC11522439; doi:10.1038/s41598-024-77306-9)
Supplement: Supplementary file 1 — Supplementary Material 1 [file 41598_2024_77306_MOESM1_ESM.docx]

**Risk Factors for Ascaris lumbricoides Infection and Its Association with Nutritional Status and IQ in 14-Year Old Adolescents in Chitwan, Nepal**

Rajendra Prasad Parajuli^1,2^*, Shristi Bhandari^1^, Lauren Marie Ward^3^, Jose Ricardo Suarez^2^

^1^ Central Department of Zoology, Tribhuvan University, Kritipur, Kathmandu, Nepal

^2^ Herbert Wertheim School of Public Health and Human Longevity Science, University of California San Diego (UCSD), California, USA

^3^Dept. of Environmental Health Sciences, University of Michigan School of Public Health, 1415 Washington Heights, Ann Arbor, MI 48109, USA

* Corresponding author: Rajendra Prasad Parajuli, PhD

Associate Professor of Zoology, Central Dept of Zoology, TU, Kathmandu, Nepal (On Sabbatical)

Research Scholar

Herbert Wertheim School of Public Health and Human Longevity Science,

University of California San Diego (UCSD), 9500 Gillman Dr, San Diego, California, USA

**E-Mails:**

RPP: [rajendra.parajuli@cdz.tu.edu.np](mailto:rajendra.parajuli@cdz.tu.edu.np), [rparajuli@health.ucsd.edu](mailto:rparajuli@health.ucsd.edu)

SB:[shristibhandari23@gmail.com](mailto:shristibhandari23@gmail.com)

LW: [lmward@umich.edu](mailto:lmward@umich.edu)

JRS: [jrsuarez@health.ucsd.edu](mailto:jrsuarez@health.ucsd.edu)

**Supplementary Table 1. Behavioural, lifestyle and nutritional status characteristics of the study participants (n = 74).**

| **Characteristics** | **Not Infected with STHs (n=62)** | **Infected with STHs (n=12)** | **p value** | **Total** |
| --- | --- | --- | --- | --- |
| **Behavioral and lifestyle characteristics** | n (%) | n (%) |  | n (%) |
| Use of soap for handwashing |  |  |  |  |
| Yes | **23 (37.1)** | **1 (8.3)** | **0.046^#^** | 24 (32.4) |
| No | **39 (62.9)** | **11 (91.7)** |  | 50 (67.6) |
| Walk barefoot while outdoors |  |  |  |  |
| No | 31 (50.0) | 7 (58.3) | NS* | 38 (51.4) |
| Yes | 31 (50) | 5 (47.3) |  | 36 (48.6) |
| Did you trim nails regularly? |  |  |  |  |
| Frequently | 20 (32.3) | 7 (58.3) | NS* | 27 (36.7) |
| Rarely | 42 (67.7) | 5 (41.7) |  | 47 (63.5) |
| Did you bite your nails? |  |  |  |  |
| No | 45 (72.6) | 6 (50.0) | NS* | 51 (68.9) |
| Yes | 17 (27.4) | 6 (50.0) |  | 23 (31.1) |
| Do you cover food regularly? |  |  |  |  |
| Yes | 42 (67.7) | 8 (67.7) | NS^#^ | 50 (67.6) |
| No | 20 (32.3) | 4 (33.3) |  | 24 (32.4) |
| Did you take anthelmintics in the last 6 months? |  |  |  |  |
| Yes | 6 (10.2) | 1 (9.1) | NS^#^ | 7 (10.0) |
| No | 53 (89.8) | 11 (90.9) |  | 63 (90.0) |
| Do you play with soil in school? |  |  |  |  |
| No | 23 (37.7) | 2 (16.7) | NS^#^ | 25 (34.2) |
| Yes | 38 (62.3) | 10 (83.3) |  | 48 (65.8) |
| What type of water do you drink? |  |  |  |  |
| Boiled or filtered | 12 (20.0) | 4 (33.3) | NS^#^ | 16 (22.2) |
| Other | 48 (80.0) | 8 (66.7) |  | 56 (77.8) |
| Do you wash raw vegetables/fruits prior to eating? |  |  |  |  |
| Yes | 50 (80.6) | 9 (75.0) | NS* | 59 (79.7) |
| No | 12 (19.4) | 3 (25.0) |  | 15 (20.3) |
| BMI Z Score Category |  |  |  |  |
| BMIZ >-2SD | 51 (83.6) | 10 (83.3) | NS^#^ | 61 (83.6) |
| BMIZ <-2SD | 10 (16.4) | 2 (16.7) |  | 12 (16.4) |

#Fisher’s exact test

* Chi-square test,

BMIZ: z scores for body mass index (BMI), SD: standard deviation, STHs: soil-transmitted helminths,

| **Supplementary Table 2. Prevalence and odds ratio of *A. lumbricoides* with respect to behavioral and individual characteristics using logistic regression analysis (n=74).** | | | |
| --- | --- | --- | --- |
|  | *A. lumbricoides* (*n* =74) | | |
|  | Univariate | | Multivariate |
|  | n (%) | OR (95%CI) | AOR (95%CI) |
| **Behavioral Lifestyle factors** |  |  |  |
| Use of soap for handwashing |  |  |  |
| Yes | **4.2** | ref | ref |
| No | **22.0** | 6.49 (0.79 to 53.56) | 5.12 (0.55 to 47.25) |
| Walk barefoot while outdoors |  |  |  |
| No | 18.4 | ref |  |
| Yes | 13.9 | 0.71 (0.20 to 2.50) |  |
| Did you trim nails regularly? |  |  |  |
| Frequently | 25.9 | ref |  |
| Rarely | 10.6 | 0.34 (0.10 to 1.21) |  |
| Did you bite your nails? |  |  |  |
| No | 11.8 | ref | ref |
| Yes | 26.1 | 2.65 (0.75 to 9.35) | 2.66 (0.72 to 9.82) |
| Do you cover food regularly? |  |  |  |
| Yes | 16.0 | ref |  |
| No | 16.7 | 1.05 (0.28 to 3.90) |  |
| Did you take anthelmintic in the last 6 months? |  |  |  |
| Yes | 14.3 | ref |  |
| No | 15.9 | 1.13 (0.12 to 10.45) |  |
| Do you play with soil in school while playing? |  |  |  |
| No | 8.0 | ref | ref |
| Yes | 20.8 | 3.03 (0.61 to 15.05) | 1.75 (0.31 to 9.86) |
| What type of water do you drink? |  |  |  |
| Boiled or filtered | 25.0 | ref |  |
| Other | 14.3 | 0.50 (0.13 to 1.94) |  |
| Do you wash raw vegetables and fruits prior to eating? |  |  |  |
| Yes | 15.3 | ref |  |
| No | 20.0 | 1.39 (0.32 to 5.93) |  |
| BMIZ Category |  |  |  |
| BMIZ > -2SD (Normal) | 16.4 | ref |  |
| BMIZ <-SD (Underweight) | 16.7 | 1.02 (0.19 to 5.38) |  |

OR: odds ratio, AOR: adjusted odds ratio, 95% CI: 95% confidence interval, %: prevalence percentage, ref: reference

BMIZ: z scores for body mass index (BMI), SD: standard deviation


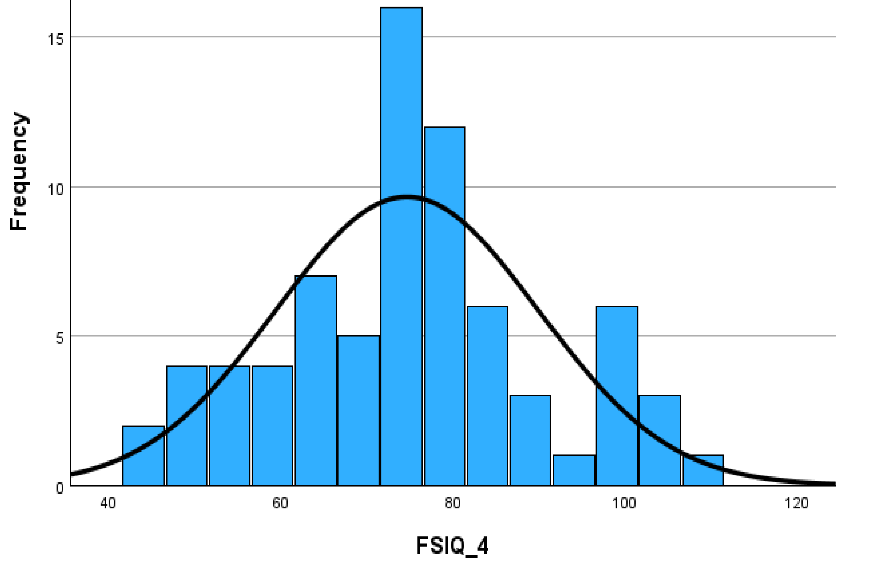


Supplementary Fig. 1. The full-scale intelligence quotient (FSI) with 4 subsets (FSIQ-4) was measured by the WASI-II, and the results are shown with a normal distribution curve.
